# Spastin MIT Domain Disease-Associated Mutations Disrupt Lysosomal Function

**DOI:** 10.3389/fnins.2019.01179

**Published:** 2019-11-08

**Authors:** Rachel Allison, James R. Edgar, Evan Reid

**Affiliations:** ^1^Department of Medical Genetics, Cambridge Institute for Medical Research, University of Cambridge, Cambridge, United Kingdom; ^2^Department of Pathology, University of Cambridge, Cambridge, United Kingdom

**Keywords:** ESCRT, endosome tubule fission, IST1, CHMP1B, hereditary spastic paraplegia

## Abstract

The hereditary spastic paraplegias (HSPs) are genetic motor neuron diseases characterized by progressive degeneration of corticospinal tract axons. Mutations in SPAST, encoding the microtubule-severing ATPase spastin, are the most common causes of HSP. The broad SPAST mutational spectrum indicates a haploinsufficiency pathogenic mechanism in most cases. Most missense mutations cluster in the ATPase domain, where they disrupt the protein's ability to sever microtubules. However, several putative missense mutations in the protein's microtubule interacting and trafficking (MIT) domain have also been described, but the pathogenicity of these mutations has not been verified with functional studies. Spastin promotes endosomal tubule fission, and defects in this lead to lysosomal enzyme mistrafficking and downstream lysosomal abnormalities. We investigated the function of three disease-associated spastin MIT mutants and found that none was able to promote normal endosomal tubule fission, lysosomal enzyme receptor trafficking, or lysosomal morphology. One of the mutations affected recruitment of spastin to endosomes, a property that requires the canonical function of the MIT domain in binding endosomal sorting complex required for transport (ESCRT)-III proteins. However, the other mutants did not affect spastin's endosomal recruitment, raising the possibility of pathologically important non-canonical roles for the MIT domain. In conclusion, we demonstrate that spastin MIT mutants cause functional abnormalities related to the pathogenesis of HSP. These mutations do not directly affect spastin's microtubule-severing capacity, and so we identify a new molecular pathological mechanism by which spastin mutations may cause disease.

## Introduction

The hereditary spastic paraplegias (HSPs) are a group of inherited neurodegenerative disorders characterized by spastic paralysis of the legs. This is caused by progressive degeneration of the longest axons of the corticospinal tract, the main motor pathway that connects the brain to the spinal cord (Harding, 1993; Reid, 1999; Fink, 2006). To date, more than 70 genes linked to HSP have been identified (Hensiek et al., 2015). Mutations in the gene encoding spastin (SPAST/SPG4; MIM:604277) are the most common cause of autosomal dominant uncomplicated “pure” HSP (MIM:182601) in North America and northern Europe, accounting for ~40% of cases (Hazan et al., 1999; Blackstone et al., 2011). The wide spectrum of SPG4 mutations, including non-sense, frameshift, splice site, and missense mutations, as well as whole exon and whole gene deletions, has led to a general consensus that the disease has a haploinsufficiency pathological mechanism in most cases, and this is supported by findings of reduced expression of spastin protein in stem cell-derived neurons or olfactory neurospheres from patients with a variety of spastin mutational classes (Abrahamsen et al., 2013; Denton et al., 2014; Havlicek et al., 2014; Rehbach et al., 2019). However, the potential for dominant negative effects or even a gain of function mechanism for some missense mutations has also been raised (Hazan et al., 1999; Fonknechten et al., 2000; Errico et al., 2002; Solowska et al., 2010, 2017).

Spastin is a microtubule-severing ATPase that uses energy from ATP hydrolysis to sever microtubules. In microtubule severing, spastin's ATPase domains associate to form a hexameric ring structure with a central pore, within which basic residues interact with the C-terminal acidic tail of tubulin (Errico et al., 2002; White et al., 2007; Roll-Mecak and Vale, 2008). ATP hydrolysis is then coupled to microtubule severing by a process that is still not fully understood, but which by analogy with structures of the related enzyme katanin may involve cycling of the hexameric complex between an open spiral and closed ring, so providing the power stroke for microtubule breakage (Roll-Mecak and Vale, 2008; Zehr et al., 2017).

**Figure 1:**
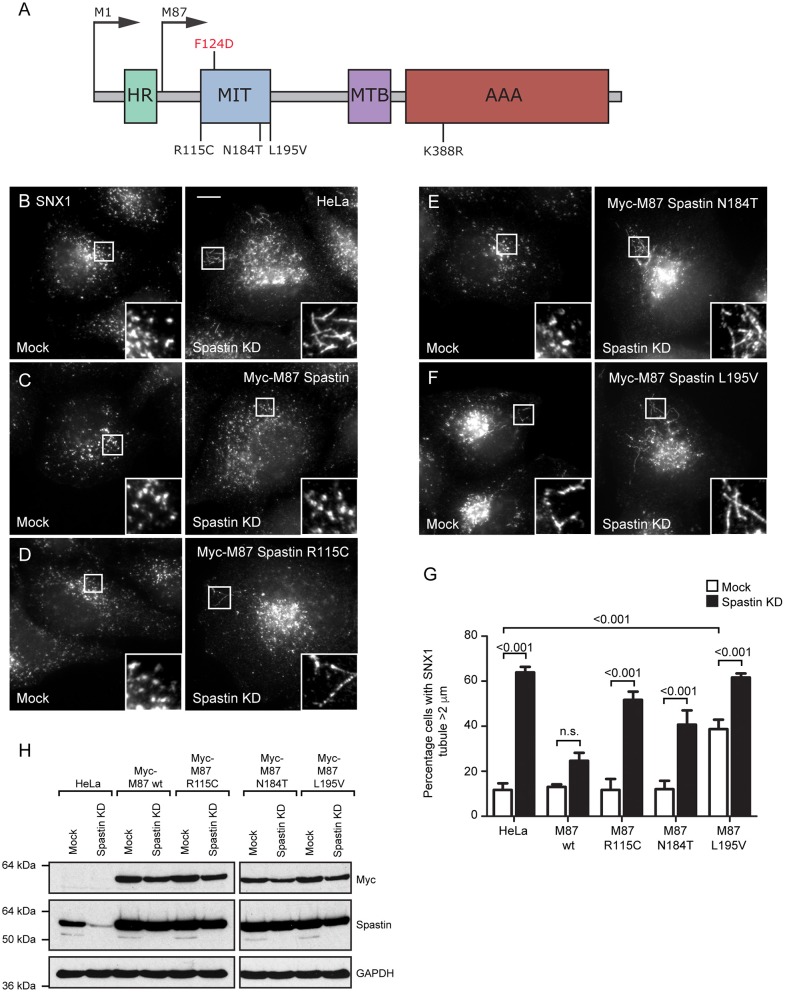
Disease-associated spastin MIT mutants cannot support normal endosomal tubule fission. **(A)** Schematic diagram of spastin's domain structure, annotated with the position of artificial (above) and disease-associated (below) mutants used in this study. HR, hydrophobic region; MIT, MIT domain; MTB, microtubule binding domain; AAA, ATPase domain. **(B–F)** Wild-type HeLa cells or cells expressing the siRNA-resistant spastin constructs indicated were subjected to mock transfection or siRNA knockdown (KD) of endogenous spastin, then fixed and labeled for endogenous SNX1. Insets show higher magnification views of the boxed areas. Scale bar = 10 μm. **(G)** The percentage of cells with the longest SNX1 tubule >2 μm was counted (*n* = 100 cells per condition). Mean and SEM are shown for three biological repeats. *P*-values were calculated using one-way ANOVA with Bonferroni's *post hoc* multiple comparison test. In addition to the *p*-values shown, the M87-wild-type KD value was significantly different from all other KD values, with a significance of at least *p* < 0.05. **(H)** Depletion of spastin and expression of siRNA resistant constructs was confirmed by Western blotting with the antibodies indicated. GAPDH labeling is shown to verify equal sample loading.

There are two major isoforms of the spastin protein, which are both ubiquitously expressed; a full-length M1 isoform and a shorter M87 isoform, generated by mechanisms involving a combination of alternative translation start sites in the full-length transcript and an alternative transcript encoding only the M87 form (Figure 1A) (Claudiani et al., 2005). At steady state, M87 spastin is mainly cytosolic, while M1-spastin predominantly localizes to the endoplasmic reticulum (ER), where it interacts with a number of other HSP proteins that are involved in ER shaping, such as REEP1 and Atlastin-1 (Sanderson et al., 2006; Connell et al., 2009; Park et al., 2010; Montenegro et al., 2012). These two isoforms of spastin appear to act in concert to drive efficient fission of tubules from the early sorting endosome, specifically at sites of contact between the endosomal tubules and the ER (Connell et al., 2009; Allison et al., 2013, 2017).

Defective endosomal tubule fission following depletion of spastin results in the missorting of receptors that traffic via this tubular-vesicular pathway, including the mannose 6-phosphate receptors (M6PRs) (Allison et al., 2013, 2017). As these receptors are not properly sorted away from early endosomes in cells lacking spastin, they remain in the endosomal compartment and traffic through to the LAMP1-positive endolysosomal degradative compartment. M6PRs normally cycle between the endosome and Golgi; upon reaching the trans-Golgi network (TGN), they capture M6P-tagged lysosomal enzymes and mediate their traffic to the endoso-lysosomal degradative compartment (Carlton et al., 2004). Thus, in cells lacking spastin, deficiency of M6PRs at the TGN causes mistrafficking of lysosomal enzymes that should be delivered from the TGN to the endolysosomal compartment (Allison et al., 2017). In turn, this causes abnormal lysosome morphology and function, characterized by increased lysosomal size, the accumulation of dense membranous material within the lysosomes, increased lysosomal acidity, and a slight reduction in lysosome numbers (Allison et al., 2017; Newton et al., 2018). The morphological abnormalities are found in lysosomes in the cell bodies and axons of human neurons derived from spastin-HSP patients via induced pluripotent stem cells (iPSCs) and in mouse primary cortical neurons from a spastin-HSP mouse model (Allison et al., 2017). The abnormal lysosomes accumulate in axonal swellings and so are compelling candidates to be involved in the pathogenesis of spastin-HSP. Similar abnormal lysosomal morphologies have also been observed in several other genetic subtypes of HSP, and we have proposed that lysosomal dysfunction is a final common disease pathway for many subtypes of HSP (Renvoise et al., 2014; Hirst et al., 2015; Allison et al., 2017).

Spastin's recruitment to endosomal membranes relies upon the interaction of its microtubule interacting and trafficking (MIT) domain with two non-canonical members of the endosomal sorting complex required for transport (ESCRT)-III, CHMP1B and IST1 (Reid et al., 2005; Agromayor et al., 2009; Yang et al., 2009; Renvoise et al., 2010). The MIT domain of spastin binds to MIT-interaction motifs (MIMs) in the C-terminal ends of CHMP1B and IST1. A functional MIT domain that is able to interact with ESCRT-III is critical for the correct regulation of endosomal tubule fission and downstream trafficking pathways by spastin, as introduction of the artificial F124D mutation into the MIT domain, which abrogates ESCRT binding and endosomal recruitment, leads to defective endosomal tubule fission, perturbed endosome-to-Golgi M6PR traffic, and abnormal lysosomal morphology (Yang et al., 2009; Allison et al., 2013, 2017).

Most missense mutations associated with spastin-HSP are in the ATPase domain and affect spastin's microtubule severing ability in a number of different ways; for example, they may block ATP binding or hydrolysis, preventing hexamerization, or disrupt the interaction between the ATPase domain and tubulin, thereby rendering the ATPase domain non-functional (White et al., 2007; Roll-Mecak and Vale, 2008). However, several families with sequence changes in the region encoding the MIT domain have also been described, although the pathogenicity or mechanism of action of such putative mutations has not been verified (Patrono et al., 2002; Crippa et al., 2006; Rudenskaia et al., 2010). In this study, we investigate the effects of several MIT domain mutants upon functions of spastin. We show that these mutations are unable to correctly regulate endosomal tubule fission, M6PR traffic, or lysosomal morphology. One of the mutations studied affected the canonical function of the MIT domain in recruitment of spastin to endosomes. However, two other mutations did not affect endosomal recruitment of spastin, indicating that non-canonical functions of the MIT domain are also important in driving endosomal tubule fission. Thus, we demonstrate that MIT mutants cause cellular abnormalities related to the pathogenesis of HSP via a novel mechanism that does not directly involve disruption of the protein's microtubule-severing activity.

## Methods

### Patient

Informed written consent was obtained to publish anonymized clinical and molecular genetic details from a patient with HSP who attended the East Anglian Regional Genetics Service. The participant also gave written informed consent to participate in the Molecular Pathology of Human Genetic Disease study, for which ethical approval was granted by the South Birmingham and East of England Cambridgeshire research ethics committees.

### Antibodies

Rabbit polyclonal anti-spastin (86–340) (raised against a glutathione S-transferase fusion protein that incorporated residues 86–340 of M1-spastin) antibodies were produced as previously described (Connell et al., 2009). Mouse monoclonal α-tubulin (DM1A) and peroxidase-conjugated secondary antibodies for Western blotting were obtained from Sigma-Aldrich; mouse monoclonal anti-myc (4A6) from EMD Millipore; mouse monoclonal anti-SNX1 (611482) from BD transduction laboratories; rabbit polyclonal anti-GFP (ab6556) and mouse monoclonal anti-M6PR (ab2733) from Abcam; mouse monoclonal anti-LAMP1 (H4A3) from Santa Cruz Biotechnology, Inc.; rabbit polyclonal anti-GAPDH (2118) from Cell Signaling Technology; and Alexa Fluor 488– and 568–labeled secondary antibodies for immunofluorescence from Molecular Probes.

### Constructs

pLXIN-myc-Spastin constructs were generated as previously described (Allison et al., 2013). In brief, M87- or M1-spastin was cloned into the pIRESneo2 vector (NheI–BamHI; Takara Bio Inc.) followed by insertion of a myc tag (EcoRV–NheI), and then myc-spastin was further cloned into the pLXIN vector (SalI–BamHI; Takara Bio Inc.). Constructs were made resistant to spastin siRNA1 and three by introducing two mutations into each of the relevant sequences by site-directed mutagenesis. Mutant versions of pLXIN-myc-M87-spastin were then generated by site-directed mutagenesis. The GFP-VPS4-E235Q construct was a gift from P. Whitley (University of Bath, Bath, England, UK).

### Generation of Stable Cell Lines

Stable cell lines were generated by retroviral transduction of HeLa cells using the spastin pLXIN constructs described above, according to previous methods (Allison et al., 2013).

### Cell Culture and Transfection

HeLa cells were maintained as previously described (Connell et al., 2009). HeLa cells stably expressing spastin constructs were additionally cultured in the presence of 500 μg/ml Geneticin/G418 (Invitrogen). This selected for cells expressing the constructs that contain a gentamycin resistance cassette. In DNA transfections, cells were transfected using Effectene Transfection Reagent (Qiagen) following the manufacturer's protocol and typically incubated with transfection reagents for 24 h. For siRNA transfection, cells were transfected with the relevant siRNAs, using Oligofectamine transfection reagent (Invitrogen), according to a protocol modified from Motley et al. (2003). In brief, cells were plated into one well of a six-well plate and transfected after 24 h. Cells were harvested 48–96 h later. The efficiency of siRNA knockdown was verified by immunoblotting cell lysates or by immunofluorescent microscopy of fixed cells, with an antibody against the relevant protein. The following siRNA sequences and concentrations were used: spastin (10 nM): siRNA1, 5′-GAACUUCAACCUUCUAUAA-3′ (Dharmacon D-014070-01); siRNA3, 5′-UAUAAGUGCUGCAAGUUUA-3′ (Dharmacon D-014070-03).

### Immunofluorescence Microscopy

Cells were fixed at room temperature (RT) in 3.8% (vol/vol) formaldehyde in phosphate buffered saline (PBS) and permeabilized in PBS containing 0.1% (vol/vol) saponin (Sigma-Aldrich) or 0.1% (vol/vol) Triton X-100 (Sigma-Aldrich). Coverslips were labeled with primary and secondary antibodies as previously described (Connell et al., 2009). Slides were analyzed with an LSM880 confocal microscope (100 × NA 1.40 oil immersion objective, 37°C), LSM780 confocal microscope (63 × NA 1.40 oil immersion objective, 37°C), or AxioImager Z2 Motorized Upright Microscope (63 × NA 1.40 oil immersion objective, RT, Axiocam 506; ZEISS), all with ZEN analysis software (ZEISS). Images were subsequently processed using ImageJ, Adobe Photoshop, Adobe Illustrator, and ZEN analysis software. Co-localization of proteins was quantified using Volocity (PerkinElmer).

### Endosomal Tubule Counts on Fixed Cells

Cells were processed for immunofluorescence microscopy and imaged with an AxioImager Motorized Upright Microscope under a 63 × /1.4-NA oil immersion objective as described earlier. Tubulation was quantified as described previously (Allison et al., 2017). Briefly, images of typically 100 cells per experimental condition were randomized and scored blind for the presence of tubules longer than 2 μm. The percentage of cells with such tubules was then calculated.

### Lysosome Quantification

The percentage of cells with large lysosomes was determined as described previously (Allison et al., 2017); fixed cells labeled with LAMP1 were processed for immunofluorescence microscopy and imaged with an AxioImager Motorized Upright Microscope. At least 100 cells were recorded per experimental condition. Images were randomized, and the largest lysosome per cell was measured using ZEN analysis software.

### Electron Microscopy

HeLa cells were grown on plastic dishes and fixed with 2% PFA, 2.5% glutaraldehyde, and 0.1 M cacodylate buffer, pH 7.2, before being scraped and spun into a pellet. All samples were postfixed with 1% osmium tetroxide:1.5% potassium ferricyanide before being incubated with 1% tannic acid to enhance contrast. Cells were dehydrated using increasing percentages of ethanol before being embedded onto EPON stubs or beam capsules. Resin was cured overnight at 65°C, and coverslips were removed using a heat-block (plastic) or repetitive freeze-thaw cycles with liquid nitrogen (glass). Ultrathin (50–70-nm) conventional sections were cut using a diamond knife mounted to a Reichart ultracut S ultramicrotome. Sections were collected onto copper grids stained using lead citrate. Sections were viewed on a FEI Tecnai transmission electron microscope at a working voltage of 80 kV.

### Yeast Two Hybrid Assay

Yeast two hybrid (Y2H) assays were carried out using the Clontech Matchmaker system as previously described (Harbour et al., 2010). Residues 110–199 of the spastin MIT domain (WT, F124D, E112K, R115C, V162I, N184T, or L195V), full-length IST1 or CHMP1B, were cloned into both pGBT9 (bait) and pGAD424 (prey), generating fusions with the GAL4 binding protein and GAL4 activation protein, respectively. The HF7c strain was used as the reporter strain with growth on plates lacking histidine (–His) as the readout, indicating that an interaction has occurred. Yeast transformations were carried out as described in Reddy and Seaman (2001).

### GST Pull-Down Assay

Residues 110–199 of the spastin MIT domain (WT, F124D, E112K, R115C, V162I, N184T, or L195V) were cloned into the BamHI-XhoI sites of pGEX-4T2 (GE Healthcare Life Sciences). The MIM domains of IST1, residues 319–364, and the single MIM of CHMP1B, residues 174–199, with the addition of a myc and a 6His tag were cloned into the BamHI-NotI sites of pGEX-6P-1 (GE Healthcare Life Sciences).

Protein was expressed in BL21(DE3)pLysS *Escherichia coli* and subsequently purified on GST sepharose as described in Collins et al. (2002). Spastin MIT domain proteins were eluted in buffer containing 10 mM glutathione (Collins et al., 2002), whereas the GST tag was removed from IST1 and CHMP1B MIMs by overnight cleavage at 4°C with Prescission protease (GE Healthcare Life Sciences) as described in the manufacturer's protocol.

GST pull-down assays were carried out as follows: 2 nmole of both bait (MIT domain) and prey (MIM) protein were bound to 20 μl packed volume of glutathione sepharose beads in 1 ml pull-down buffer (PBS + 0.1% NP-40 + 1 mM DTT) overnight at 4°C with rotation. Pull-downs were then washed three times in ice-cold pull-down buffer and resuspended in 30 μl of 3x protein sample buffer. Samples were then separated on an 18% Tris-Tricine gel at 120 V for 3 h in duplicate, half of each pull-down Coomassie stained to assess total protein, and the other half subject to Western blotting and subsequent detection of prey protein bands with anti-myc antibody (Millipore).

### Statistical Analysis

Statistical analyses were performed by paired two-tailed *t*-tests or one-way ANOVA with Bonferroni's (when multiple treatments were compared against each other) or Dunnett's (when multiple treatments were compared to a single control value) *post hoc* tests for multiple comparisons tests, using GraphPad Prism version 5.01 for Windows, GraphPad Software, La Jolla California, USA (www.graphpad.com).

## Results

### Disease-Associated MIT Domain Mutants Cannot Regulate Endosomal Tubulation

Three putative mutations in the MIT domain of spastin were identified from the existing literature, E112K, N184T, and L195V (Patrono et al., 2002; Crippa et al., 2006; Rudenskaia et al., 2010). All were found in patients with pure HSP. A novel MIT domain mutation was also identified in a patient attending the East Anglian Medical Genetics Service, with a phenotype consistent with pure HSP and no family history of spastic paraplegia, R115C (SPAST c.343C>T). In each case, limited segregation data were available, but the sequence changes each affect moderately to highly evolutionarily conserved amino acids and are not present, or in the case of R115C only present once, in the gnomAD database of several hundred thousand alleles (Lek et al., 2016). SIFT and Polyphen pathogenicity predictions for each variant, generated via the Ensemble Variant Effect Predictor, are shown in Table 1 (McLaren et al., 2016).

**Table 1:** Results of pathogenicity prediction programs for MIT domain sequence changes identified in HSP patients.

**Mutation**	**SIFT prediction (score)**	**PolyPhen prediction (score)**
E112K	Tolerated (0.74)	Benign (0.017)
R115C	Deleterious (0.02)	Benign (0.251)
N184T	Deleterious (0)	Probably damaging (1)
L195V	Deleterious (0)	Possibly damaging (0.823)

The MIT domain consists of three α helices that form an anti-parallel up and down helix bundle fold, forming a MIM binding region in between helices α1 and α3 (Yang et al., 2009). R115C and E112K lie at the N-terminus of the MIT domain, in the linker region adjoining helix α1, and L195V is at the very C-terminus of helix α3. N184T lies within the MIM binding region on α3 and is one of the residues involved in MIT-CHMP1B MIM interaction (Figure 1A) (Yang et al., 2009). We generated constructs encoding forms of spastin containing each mutant. We began by confirming that each retained the ability to sever microtubules, which we tested by transiently expressing in HeLa cells and then assessing the microtubule architecture by immunofluorescence microscopy (Supplementary Figures 1A–F). Each of the mutants exhibited a similar degree of microtubule depletion, which was comparable to that of wild-type spastin and distinct from the microtubule bundling phenotype that has been observed in some missense mutations that block ATP binding or hydrolysis (e.g., K388R; Figure 1A; Supplementary Figure 1C) (Errico et al., 2002).

We next examined whether the patient MIT mutants affected spastin's role in endosomal tubule fission. Spastin requires the ability to bind ESCRT-III and to hydrolyze ATP for this function, as previous siRNA rescue experiments showed that forms of spastin containing either the artificial F124D MIT mutation (which blocks ESCRT-III interaction) or the disease-associated K388R mutation (which blocks ATP hydrolysis), failed to rescue the increased tubulation of sorting nexin 1 (SNX1; marker of a subset of endosomal tubules) seen in cells lacking endogenous spastin (Allison et al., 2013). We investigated the ability of the disease-associated MIT mutants to promote endosomal tubule fission using siRNA-rescue experiments with cells stably expressing siRNA-resistant forms of M87-spastin harboring each of the mutants (except the E112K mutant, as repeated attempts to generate a stable cell line failed). Stable expression of the patient mutants did not affect the cellular microtubule architecture (Supplementary Figures 1G–K). The increased SNX1 tubulation phenotype seen in cells lacking endogenous spastin was rescued by wild-type siRNA-resistant spastin but not by any of the patient MIT mutant forms (Figures 1B–H). As the spastin-depleted cells lack both endogenous M1- and M87-spastin, this effect of the mutants cannot be via a dominant negative effect on wild-type M1-spastin, as has been proposed as a mechanism for some mutations that lie outside of the ATPase domain (Solowska et al., 2010). We concluded that the amino acids affected by the patient mutations are essential for spastin's role in promoting endosomal tubule fission.

### Mutations in Spastin's MIT Domain Disrupt Endosome-To-Golgi Traffic

**Figure 2:**
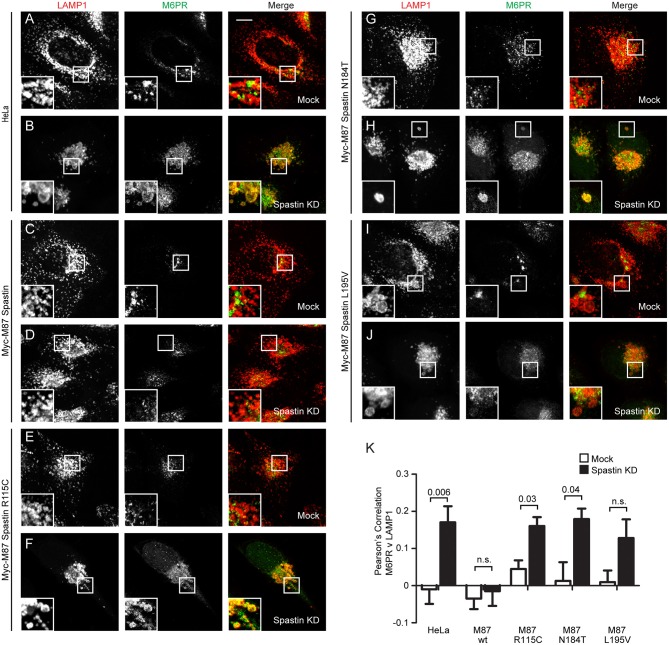
Endosome to Golgi M6PR traffic is disrupted by MIT domain mutations in spastin. **(A–J)** Wild-type HeLa cells or cells expressing the siRNA-resistant spastin constructs indicated were subjected to mock transfection or siRNA-mediated KD of endogenous spastin, then processed for immunofluorescence microscopy and labeled with antibodies to M6PR and the lysosomal marker LAMP1. Insets show higher magnification views of the boxed areas. Scale bar = 10 μm. **(K)** Co-localization between M6PR and LAMP1 was quantified by calculating the Pearson's correlation coefficient for red and green pixels in each cell using Volocity software (20 cells per condition quantified in each experiment). Mean and SEM are shown for *n* = 4 experiments. *P*-values were calculated using paired two-tailed *t*-tests.

In cells depleted of spastin, defective endosomal tubule fission causes failure of M6PR to sort away from the endosome, resulting in its accumulation in a LAMP1-positive endolysosomal compartment (Allison et al., 2017). This phenotype can be rescued by wild-type spastin but not by forms unable to bind ESCRT-III or hydrolye ATP (Allison et al., 2017). We examined whether the disease-associated MIT mutants were able to rescue this mislocalization of M6PR. Following siRNA-mediated spastin depletion in wild-type HeLa cells, we observed a significant increase in the co-localization of LAMP1 and M6PR, which as expected was rescued by expression of wild-type myc-M87 spastin (Figures 2A–D,K). However, none of the MIT mutant forms rescued this phenotype (Figures 2E–K). Disease-associated mutations in spastin's MIT domain are therefore unable to support normal sorting of M6PR from the endosome to the Golgi, consistent with their inability to drive efficient endosomal tubule fission.

### Mutations in Spastin's MIT Domain Lead to Abnormal Lysosome Morphology

**Figure 3:**
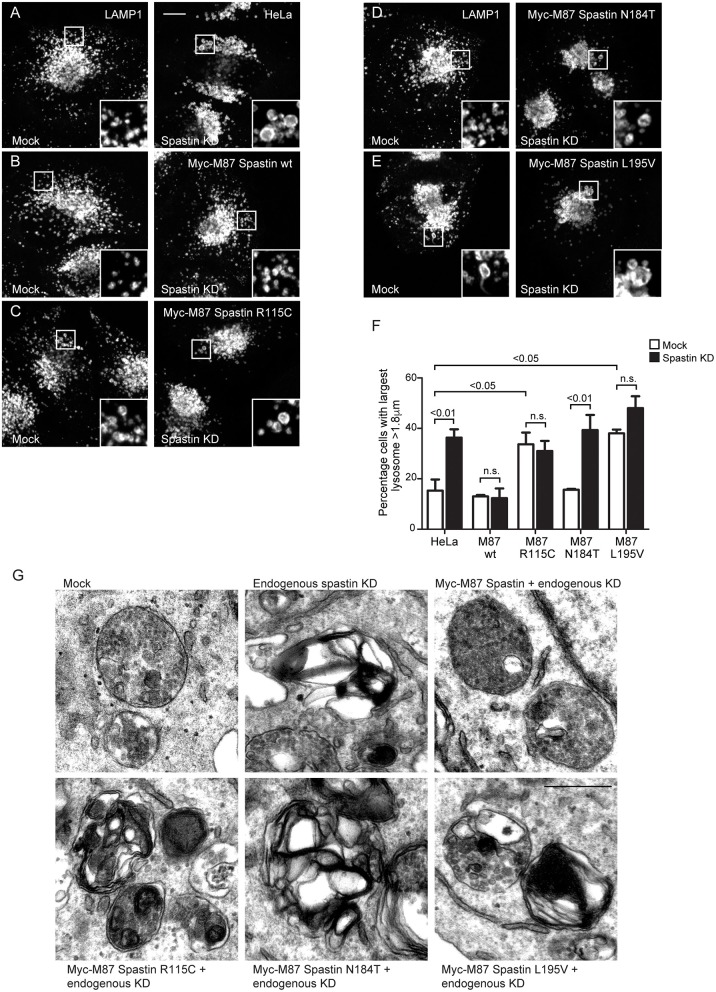
Spastin MIT domain mutants cannot support normal lysosomal morphology. **(A–E)** Wild-type HeLa cells or cells expressing the siRNA-resistant spastin constructs indicated were subjected to mock transfection or siRNA-mediated KD of endogenous spastin, then processed for immunofluorescence microscopy and labeled for LAMP1. Insets show higher magnification views of the boxed areas. Scale bar = 10 μm. **(F)** The diameter of the largest lysosome was measured in 100 cells per condition. The mean and SEM of the percentage of cells with largest lysosome >1.8 μm is shown (*n* = 4 biological repeats). *P*-values were calculated by one-way ANOVA with Bonferroni's *post hoc* multiple comparison test. In addition to the *p*-values shown, the M87 wild-type KD value was significantly different from all other KD values with a significance of at least *p* < 0.01. **(G)** Wild-type HeLa cells or HeLa cells expressing the siRNA-resistant spastin constructs indicated were subjected to mock transfection or siRNA-mediated KD of endogenous spastin. Electron micrographs of representative endolysosomal structures are shown. Note the presence of highly abnormal structures in each of the cells expressing mutant spastin. Scale bar = 500 nm.

The abnormal M6PR traffic seen in HeLa cells lacking spastin causes abnormal lysosomal enzyme delivery, resulting in the development of enlarged lysosomes (Allison et al., 2017), associated with increased cellular abundance and altered migration of LAMP1 on immunoblotting (Supplementary Figure 2). We interrogated the ability of the MIT mutants to rescue the lysosomal enlargement phenotype with siRNA-rescue experiments and found that lysosomal enlargement was rescued by wild-type M87-spastin but not by any of the MIT mutant forms (Figures 3A–F). Two of the mutants, R115C and L195V, also had a significant dominant negative effect on lysosomal size in cells not depleted for endogenous spastin (Figures 3C,E,F).

Lysosomes in spastin-HSP cellular models have a characteristic ultrastructure, including the development of thick membrane whorls and dense honeycomb networks of membrane. We investigated the effects of the MIT mutants on lysosome ultrastructure. As expected, upon spastin depletion, we observed lysosomes with the characteristic abnormal appearances (Allison et al., 2017). This phenotype was rescued by wild-type M87 spastin but not by any of the MIT mutants (Figure 3G). The residues affected by the patient mutations are therefore required for spastin's role in maintaining correct lysosome morphology.

### Spastin MIT Domain Mutants Show Differential Recruitment to Endosomes

**Figure 4:**
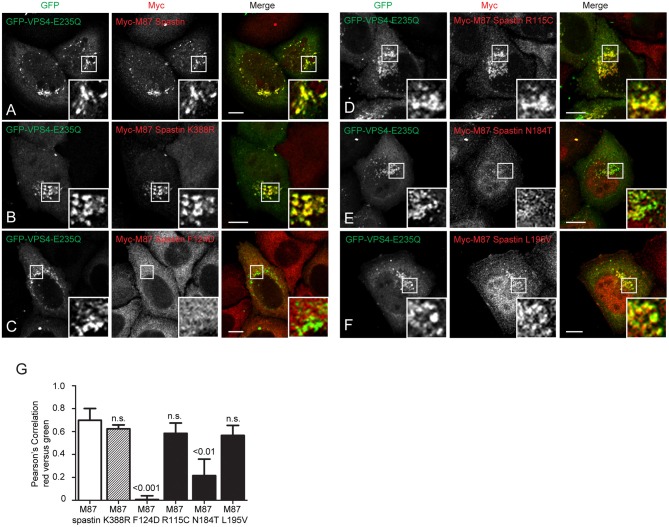
MIT mutant forms of spastin show differential recruitment to VPS4-E235Q endosomes. GFP-VPS4-E235Q was transiently transfected into cell lines stably expressing myc-tagged wild-type spastin **(A)** or spastin mutants **(B–F)**, and then cells were fixed and labeled with an anti-myc antibody. Insets show higher magnification views of the boxed areas. **(G)** Co-localization between GFP-VPS4-E235Q and spastin proteins was estimated by calculating the Pearson's correlation coefficient for red and green pixels in each cell, using Volocity software (*n* = 4 experiments, 15 cells per condition quantified in each experiment). Mean and SEM are shown, *p*-values vs. M87 wild-type spastin were calculated using one-way ANOVA with Dunnett's multiple comparison posttest. Scale bar = 10 μm.

We next sought to understand the mechanism by which the MIT mutants might affect spastin's function in endosomal tubule fission. At steady state, M87-spastin is mostly cytosolic and shows minimal association with endosomal markers. The canonical role of the MIT domain is to recruit spastin to endosomes via interactions with ESCRT-III, and this can be reported by expression of a dominant negative form of VPS4 (VPS4-E235Q). VPS4 normally acts to remove ESCRT-III proteins from endosomes, while the dominant negative form traps ESCRT-III proteins and associated proteins, including spastin, on the endosomal membrane, so revealing transient endosomal localizations (Stuchell-Brereton et al., 2007; Connell et al., 2009; Allison et al., 2013). Interestingly, the patient MIT mutants showed differential recruitment to GFP-VPS4-E235Q endosomes. Spastin-R115C and spastin-L195V showed no significant reduction in recruitment when compared to wild-type M87 spastin, while spastin-N184T had significantly reduced co-localization with GFP-VPS4-E235Q. As expected, endosomal recruitment of the ATPase mutant K388R was not reduced, while recruitment of the artificial F124D MIT domain mutant was almost completely abolished (Figures 4A–G). These experiments suggest that, at least for the N184T mutant, altered endosomal recruitment may account for the inability to rescue the tubulation phenotype. However, these data also indicate that the L195V and R115C mutants likely affect tubule fission by a mechanism independent of spastin's ability to recruit to endosomes.

**Figure 5:**
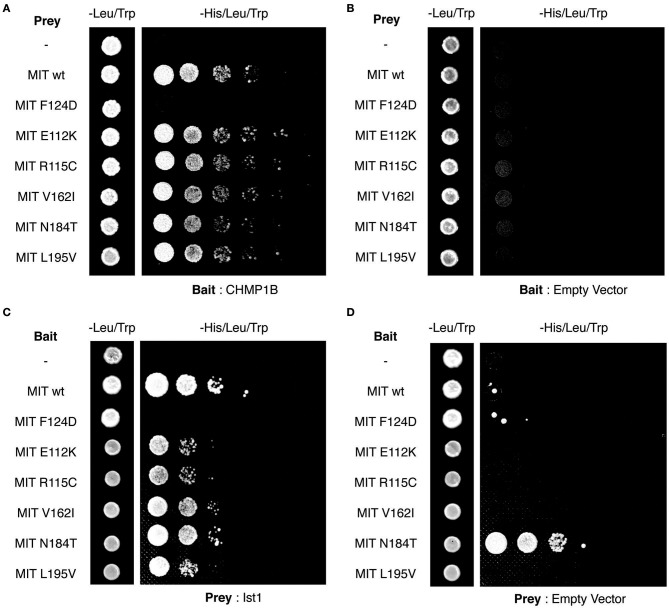
Yeast two-hybrid analysis of spastin MIT domain binding to CHMP1B and IST1. **(A–D)** Interactions between the combinations of MIT domain, CHMP1B, and IST1 bait and prey vectors indicated in each panel. In each case, the first column shows the growth of diploid colonies on media lacking leucine and tryptophan, to select for the presence of both bait and prey vectors, while columns 2–6 show the growth of serial dilutions of diploid colonies on media lacking leucine, tryptophan, and histidine, which also require the activation of the His reporter gene that occurs on interaction of bait and prey proteins. In **(B,D)**, empty vector controls test for auto-activation of by the MIT mutant proteins under investigation. Note that the N184T mutant causes auto-activation, and so binding results for this mutant were uninterpretable.

**Figure 6:**
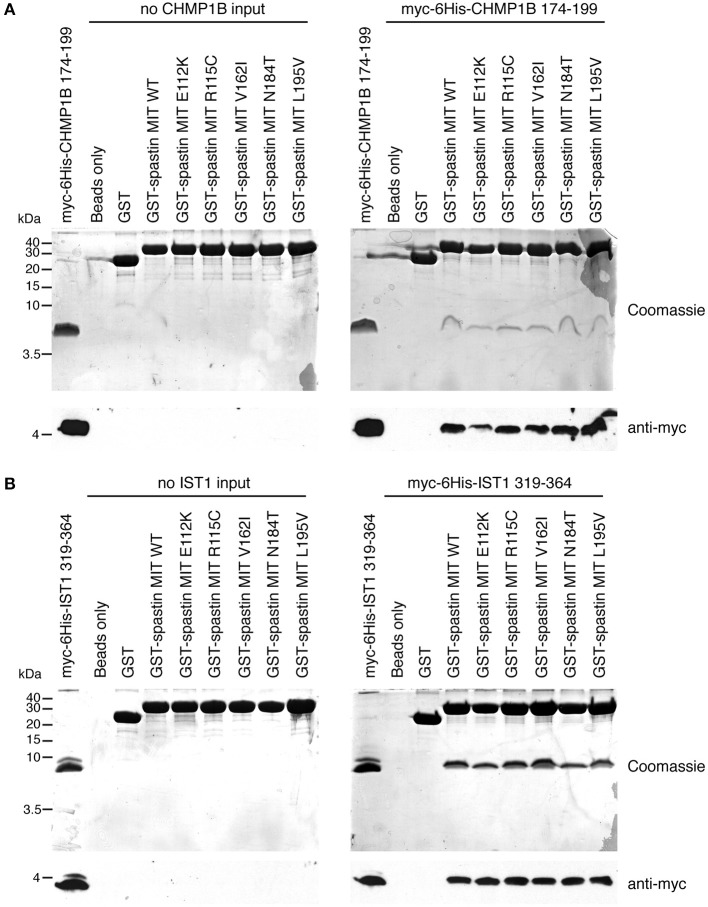
Assessment of interactions between spastin MIT domains and MIM-regions of CHMP1B and IST1 by GST pull-down. Purified proteins consisting of GST alone, GST fused to the wild-type spastin MIT domain, or to spastin MIT domains harboring the sequence changes indicated were used in *in vitro* GST pull-down experiments vs. myc- and 6His-tagged fragments of CHMP1B **(A)** or IST1 **(B)** that incorporate the spastin-binding MIM regions. In each set of experiments, the upper panels show Coomassie-stained gels, while the bottom panel shows corresponding anti-myc immunoblotting.

Finally, we tested directly whether the MIT mutants affect binding of the spastin MIT domain to IST1 or CHMP1B. We first examined this by yeast two-hybrid, where as expected the artificial F124D MIT mutant showed no interaction with CHMP1B or IST1 (Figures 5A–D). Unfortunately, auto-activation of the N184T mutant in this assay meant that its binding to the ESCRT proteins could not be assessed. However, there was no clear reduction in binding of any of the other patient MIT mutants, which exhibited interactions comparable to the wild-type MIT domain and to a known benign spastin polymorphism, V162I, which has an allele frequency of 2.22 × 10^−3^ in the general population (Lek et al., 2016). We next examined binding using GST pull-downs, testing the ability of purified wild-type and mutant spastin MIT domains to interact with regions containing the MIMs of CHMP1B (amino acids 174–199) and IST1 (amino acids 319–364) (Agromayor et al., 2009; Bajorek et al., 2009; Yang et al., 2009). As expected, there was no binding between the MIT domain harboring the F124D artificial mutant and the ESCRT MIMs (data not shown). Furthermore, in this system interaction was abolished (in the case of CHMP1B) or reduced (in the case of IST1) by introduction of another artificial MIT mutant (F124A) that is known to significantly diminish, but not completely inhibit, binding of spastin's MIT domain to CHMP1B (data not shown) (Yang et al., 2009). In contrast, there was no appreciable difference between the MIM-binding capacity of the wild-type spastin MIT, an MIT containing the benign polymorphism V162I, or any of the spastin MIT mutants, including N184T (Figures 6A,B). Thus, the abnormal functional effects of the mutants do not appear to be related to their capacity to bind CHMP1B or IST1.

## Discussion

In this study, we have shown that three patient-associated missense changes in the MIT domain affect the function of spastin, rendering it unable to correctly regulate endosomal tubule fission, with consequent effects on endosome-to-Golgi trafficking of M6PRs and lysosomal morphology. The lysosomal abnormalities are likely to be pathologically relevant as very similar lysosomal morphologies are observed in neurons from a spastin-HSP mouse model and from patient-derived neurons, including in concentrations within pathological axonal swellings (Allison et al., 2017). Considered together with the absence, or in the case of R115C very low frequency, of these mutations from general population databases, we conclude that these missense changes are likely to be pathogenic. As these mutations do not directly affect spastin's microtubule-severing capacity, coupling of these MIT mutations to lysosomal dysfunction represents a new molecular pathological mechanism by which spastin mutations may cause disease. A key challenge now is to understand how lysosomal abnormality causes axonopathy—numerous possibilities require exploration. Functional lysosomes are required for autophagy and mitophagy, with defects in these processes linked to axonopathy (Ashrafi et al., 2014; Gowrishankar et al., 2015; Xie et al., 2015). They are also required for lipid metabolism, and altered lipid metabolism appears sufficient to cause HSP (Boutry et al., 2019). Finally, lysosomes are key sites of receptor degradation, and upregulated bone morphogenetic protein signaling caused by defective receptor degradation is implicated in HSP pathogenesis (Wang et al., 2007; Tsang et al., 2009; Blackstone et al., 2011).

Of the mutations that we investigated in this study, only the N184T change caused a large reduction (>50%) in the endosomal recruitment of spastin, as compared to the wild-type protein. Thus, in the case of this mutation, defects in endosomal tubule fission, with consequent downstream defects on lysosome function, are probably caused by insufficient endosomal spastin. However, although structural studies of the binding of the MIT domain to CHMP1B show that this mutation lies within the MIM binding site and directly participates in MIM binding, surprisingly, it did not appear to affect MIT-MIM binding when assayed by GST pull-down (Yang et al., 2009). Spastin's recruitment to endosomes requires its MIT domain, but it is not known whether CHMP1B and IST1 are the only endosomal proteins involved in this recruitment. The simplest interpretation of our data is that another, perhaps novel, ESCRT-like protein in addition to IST1 and CHMP1B participates in spastin's recruitment to endosomes, and that it is this binding that is affected by the N184T alteration.

The other two mutants studied caused no reduction in endosomal recruitment, consistent with their location outside of the MIM-binding region of the spastin MIT domain. This suggests that they are affecting a non-canonical function of the MIT domain that is independent of ESCRT-III interaction and invites the possibility that non-canonical functions of the MIT domain are important for the correct function of spastin in endosomal tubule fission. Although non-canonical functions of the spastin MIT domain have not been elucidated, other MIT domains have been proposed to have Ca^2+^ or phospholipid binding properties (Iwaya et al., 2013). Interestingly, both of these mutations showed dominant negative effects on lysosomal size when stably expressed in HeLa cells. We suggest that in the physiological situation in HSP patients, when these alleles are expressed together with a wild-type allele, the mutant proteins are likely incorporated into, and negatively affect the function of, the spastin hexamer at the endosome.

In summary, by showing that MIT domain mutants do not directly affect microtubule severing, but still have pathologically relevant functional effects, we have identified a novel molecular pathological mechanism by which alteration of spastin may cause HSP. Our work hints at the existence of novel factors that may determine recruitment of spastin to endosomes, as well as indicating that interference with non-canonical functions of the MIT domain may cause the disease.

## Data Availability Statement

The raw data supporting the conclusions of this manuscript will be made available by the authors, without undue reservation, to any qualified researcher.

## Ethics Statement

The studies involving human participants were reviewed and approved by East of England Cambridgeshire research ethics committee. The patients/participants provided their written informed consent to participate in this study. Written informed consent was obtained from the individual(s) for the publication of any potentially identifiable images or data included in this article.

## Author's Note

An additional description of a family with an N184T spastin mutation was recently published (Kadnikova et al., 2019).

## Author Contributions

RA performed experiments, analyzed the data, helped direct the research, and helped write the article. JE performed experiments, analyzed the data, and helped write the article. ER directed the research, analyzed the data, and wrote the article.

### Conflict of Interest

The authors declare that the research was conducted in the absence of any commercial or financial relationships that could be construed as a potential conflict of interest.

## Supplementary Material

Supplementary Figure 1 Spastin's microtubule severing activity is unaffected by MIT mutants. **(A–F)** Wild-type Hela cells were transiently transfected with the myc-tagged M87-spastin constructs indicated. Cells were fixed and then processed for immunofluorescence microscopy and labeled with antibodies to myc and α-tubulin. Representative images showing the appearance of the microtubule architecture following spastin overexpression are shown. Scale bar = 10 μm. **(G–K)** Wild-type Hela cells or cells stably expressing the myc-tagged M87-spastin constructs indicated were fixed, then processed for immunofluorescence microscopy and labeled with **(G)** spastin and α-tubulin or **(H–K)** myc and α-tubulin. Representative images showing the appearance of microtubule cytoskeleton in each cell line, with and without endogenous spastin depletion, are shown. Scale bars = 10 μm.

Supplementary Figure 2 Cells lacking spastin exhibit upregulation and altered migration of LAMP1. **(A)** HeLa cells were mock-transfected or subjected to spastin depletion by siRNA transfection, then immunoblotted with the antibodies shown. Note increased abundance and altered LAMP1 migration in the immunoblot from cells lacking spastin. Quantification of LAMP1 immunoblot band intensity is shown in **(B)**, *n* = 4, *p*-values generated by paired *t*-tests.

## References

[B1] Abrahamsen G. Fan Y. Matigian N. Wali G. Bellette B. Sutharsan R.. (2013). A patient-derived stem cell model of hereditary spastic paraplegia with SPAST mutations. Dis. Model. Mech. 6, 489–502. 10.1242/dmm.010884 23264559 PMC3597030

[B2] Agromayor M. Carlton J. G. Phelan J. P. Matthews D. R. Carlin L. M. Ameer-Beg S.. (2009). Essential role of hIST1 in cytokinesis. Mol. Biol. Cell. 20, 1374–1387. 10.1091/mbc.e08-05-0474 19129480 PMC2649264

[B3] Allison R. Edgar J. R. Pearson G. Rizo T. Newton T. Günther S.. (2017). Defects in ER–endosome contacts impact lysosome function in hereditary spastic paraplegia. J. Cell Biol. 216, 1337–1355. 10.1083/jcb.201609033 28389476 PMC5412567

[B4] Allison R. Lumb J. H. Fassier C. Connell J. W. Ten Martin D. Seaman M. N.. (2013). An ESCRT-spastin interaction promotes fission of recycling tubules from the endosome. J. Cell Biol. 202, 527–543. 10.1083/jcb.201211045 23897888 PMC3734076

[B5] Ashrafi G. Schlehe J. S. LaVoie M. J. Schwarz T. L. (2014). Mitophagy of damaged mitochondria occurs locally in distal neuronal axons and requires PINK1 and Parkin. J. Cell Biol. 206, 655–670. 10.1083/jcb.201401070 25154397 PMC4151150

[B6] Bajorek M. Morita E. Skalicky J. J. Morham S. G. Babst M. Sundquist W. I. (2009). Biochemical analyses of human IST1 and its function in cytokinesis. Mol. Biol. Cell. 20, 1360–1373. 10.1091/mbc.e08-05-0475 19129479 PMC2649257

[B7] Blackstone C. O'Kane C. J. Reid E. (2011). Hereditary spastic paraplegias: membrane traffic and the motor pathway. Nat. Rev. Neurosci. 12, 31–42. 10.1038/nrn2946 21139634 PMC5584382

[B8] Boutry M. Morais S. Stevanin G. (2019). Update on the genetics of spastic paraplegias. Curr. Neurol. Neurosci. Rep. 19:18. 10.1007/s11910-019-0930-2 30820684

[B9] Carlton J. Bujny M. Peter B. J. Oorschot V. M. Rutherford A. Mellor H.. (2004). Sorting nexin-1 mediates tubular endosome-to-TGN transport through coincidence sensing of high- curvature membranes and 3-phosphoinositides. Curr. Biol. 14, 1791–1800. 10.1016/j.cub.2004.09.077 15498486

[B10] Claudiani P. Riano E. Errico A. Andolfi G. Rugarli E. I. (2005). Spastin subcellular localization is regulated through usage of different translation start sites and active export from the nucleus. Exp. Cell Res. 309, 358–369. 10.1016/j.yexcr.2005.06.009 16026783

[B11] Collins B. M. McCoy A. J. Kent H. M. Evans P. R. Owen D. J. (2002). Molecular architecture and functional model of the endocytic AP2 complex. Cell. 109, 523–535. 10.1016/S0092-8674(02)00735-3 12086608

[B12] Connell J. W. Lindon C. Luzio J. P. Reid E. (2009). Spastin couples microtubule severing to membrane traffic in completion of cytokinesis and secretion. Traffic. 10, 42–56. 10.1111/j.1600-0854.2008.00847.x 19000169 PMC2709849

[B13] Crippa F. Panzeri C. Martinuzzi A. Arnoldi A. Redaelli F. Tonelli A.. (2006). Eight novel mutations in SPG4 in a large sample of patients with hereditary spastic paraplegia. Arch. Neurol. 63, 750–755. 10.1001/archneur.63.5.750 16682546

[B14] Denton K. R. Lei L. Grenier J. Rodionov V. Blackstone C. Li X. J. (2014). Loss of spastin function results in disease-specific axonal defects in human pluripotent stem cell-based models of hereditary spastic paraplegia. Stem Cells. 32, 414–423. 10.1002/stem.1569 24123785 PMC3947148

[B15] Errico A. Ballabio A. Rugarli E. I. (2002). Spastin, the protein mutated in autosomal dominant hereditary spastic paraplegia, is involved in microtubule dynamics. Hum. Mol. Genet. 11, 153–163. 10.1093/hmg/11.2.153 11809724

[B16] Fink J. K. (2006). Hereditary spastic paraplegia. Curr. Neurol. Neurosci. Rep. 6, 65–76. 10.1007/s11910-996-0011-1 16469273

[B17] Fonknechten N. Mavel D. Byrne P. Davoine C. S. Cruaud C. Bonsch D.. (2000). Spectrum of SPG4 mutations in autosomal dominant spastic paraplegia. Hum. Mol. Genet. 9, 637–644. 10.1093/hmg/9.4.637 10699187

[B18] Gowrishankar S. Yuan P. Wu Y. Schrag M. Paradise S. Grutzendler J.. (2015). Massive accumulation of luminal protease-deficient axonal lysosomes at Alzheimer's disease amyloid plaques. Proc. Natl. Acad. Sci. U.S.A. 112, E3699–E708. 10.1073/pnas.1510329112 26124111 PMC4507205

[B19] Harbour M. E. Breusegem S. Y. Antrobus R. Freeman C. Reid E. Seaman M. N. (2010). The cargo-selective retromer complex is a recruiting hub for protein complexes that regulate endosomal tubule dynamics. J. Cell Sci. 123(Pt 21), 3703–3717. 10.1242/jcs.071472 20923837 PMC2964111

[B20] Harding A. E. (1993). Hereditary spastic paraplegias. Semin. Neurol. 13, 333–336. 10.1055/s-2008-1041143 8146482

[B21] Havlicek S. Kohl Z. Mishra H. K. Prots I. Eberhardt E. Denguir N.. (2014). Gene dosage-dependent rescue of HSP neurite defects in SPG4 patients' neurons. Hum. Mol. Genet. 23, 2527–2541. 10.1093/hmg/ddt644 24381312 PMC3990156

[B22] Hazan J. Fonknechten N. Mavel D. Paternotte C. Samson D. Artiguenave F.. (1999). Spastin, a new AAA protein, is altered in the most frequent form of autosomal dominant spastic paraplegia. Nat. Genet. 23, 296–303. 10.1038/15472 10610178

[B23] Hensiek A. Kirker S. Reid E. (2015). Diagnosis, investigation and management of hereditary spastic paraplegias in the era of next-generation sequencing. J. Neurol. 272, 1601–1612. 10.1007/s00415-014-7598-y PMC4503825 25480570

[B24] Hirst J. Edgar J. R. Esteves T. Darios F. Madeo M. Chang J.. (2015). Loss of AP-5 results in accumulation of aberrant endolysosomes: defining a new type of lysosomal storage disease. Hum. Mol. Genet. 24, 4984–4996. 10.1093/hmg/ddv220 26085577 PMC4527494

[B25] Iwaya N. Takasu H. Goda N. Shirakawa M. Tanaka T. Hamada D.. (2013). MIT domain of Vps4 is a Ca2+-dependent phosphoinositide-binding domain. J. Biochem. 153, 473–481. 10.1093/jb/mvt012 23423459

[B26] Kadnikova V. A. Rudenskaya G. E. Stepanova A. A. Sermyagina I. G. Ryzhkova O. P. (2019). Mutational spectrum of Spast (Spg4) and Atl1 (Spg3a) genes in Russian patients with hereditary spastic paraplegia. Sci. Rep. 9:14412. 10.1038/s41598-019-50911-9 31594988 PMC6783457

[B27] Lek M. Karczewski K. J. Minikel E. V. Samocha K. E. Banks E. Fennell T.. (2016). Analysis of protein-coding genetic variation in 60,706 humans. Nature. 536:285–91. 10.1038/nature19057 27535533 PMC5018207

[B28] McLaren W. Gil L. Hunt S. E. Riat H. S. Ritchie G. R. S. Thormann A.. (2016). The ensembl variant effect predictor. Genome Biol. 17:122. 10.1186/s13059-016-0974-4 27268795 PMC4893825

[B29] Montenegro G. Rebelo A. P. Connell J. Allison R. Babalini C. D'Aloia M.. (2012). Mutations in the ER-shaping protein reticulon 2 cause the axon-degenerative disorder hereditary spastic paraplegia type 12. J. Clin. Invest. 122, 538–544. 10.1172/JCI60560 22232211 PMC3266795

[B30] Motley A. Bright N. A. Seaman M. N. Robinson M. S. (2003). Clathrin-mediated endocytosis in AP-2-depleted cells. J. Cell Biol. 162, 909–918. 10.1083/jcb.200305145 12952941 PMC2172830

[B31] Newton T. Allison R. Edgar J. R. Lumb J. H. Rodger C. E. Manna P. T.. (2018). Mechanistic basis of an epistatic interaction reducing age at onset in hereditary spastic paraplegia. Brain. 141, 1286–1299. 10.1093/brain/awy034 29481671 PMC5917785

[B32] Park S. H. Zhu P. P. Parker R. L. Blackstone C. (2010). Hereditary spastic paraplegia proteins REEP1, spastin, and atlastin-1 coordinate microtubule interactions with the tubular ER network. J. Clin. Invest. 120, 1097–1110. 10.1172/JCI40979 20200447 PMC2846052

[B33] Patrono C. Casali C. Tessa A. Cricchi F. Fortini D. Carrozzo R.. (2002). Missense and splice site mutations in SPG4 suggest loss-of-function in dominant spastic paraplegia. J. Neurol. 249, 200–205. 10.1007/PL00007865 11985387

[B34] Reddy J. V. Seaman M. N. (2001). Vps26p, a component of retromer, directs the interactions of Vps35p in endosome-to-Golgi retrieval. Mol. Biol. Cell. 12, 3242–3256. 10.1091/mbc.12.10.3242 11598206 PMC60170

[B35] Rehbach K. Kesavan J. Hauser S. Ritzenhofen S. Jungverdorben J. Schüle R.. (2019). Multiparametric rapid screening of neuronal process pathology for drug target identification in HSP patient-specific neurons. Sci. Rep. 9:9615. 10.1038/s41598-019-45246-4 31270336 PMC6610147

[B36] Reid E. (1999). The hereditary spastic paraplegias. J. Neurol. 246, 995–1003. 10.1007/s004150050503 10631629

[B37] Reid E. Connell J. Edwards T. L. Duley S. Brown S. E. Sanderson C. M. (2005). The hereditary spastic paraplegia protein spastin interacts with the ESCRT-III complex-associated endosomal protein CHMP1B. Hum. Mol. Genet. 14, 19–38. 10.1093/hmg/ddi003 15537668

[B38] Renvoise B. Chang J. Singh R. Yonekawa S. FitzGibbon E. J. Mankodi A.. (2014). Lysosomal abnormalities in hereditary spastic paraplegia types SPG15 and SPG11. Ann. Clin. Transl. Neurol. 1, 379–389. 10.1002/acn3.64 24999486 PMC4078876

[B39] Renvoise B. Parker R. L. Yang D. Bakowska J. C. Hurley J. H. Blackstone C. (2010). SPG20 protein spartin is recruited to midbodies by ESCRT-III protein Ist1 and participates in cytokinesis. Mol. Biol. Cell. 21, 3293–3303. 10.1091/mbc.e09-10-0879 20719964 PMC2947466

[B40] Roll-Mecak A. Vale R. D. (2008). Structural basis of microtubule severing by the hereditary spastic paraplegia protein spastin. Nature 451, 363–367. 10.1038/nature06482 18202664 PMC2882799

[B41] Rudenskaia G. E. Sermiagina I. G. Illarioshkin S. N. Sidorova O. P. Fedotov V. P. Poliakov A. V. (2010). [Hereditary spastic paraplegia type 4 (SPG4): clinical and molecular-genetic characteristics]. Zh. Nevrol. Psikhiatr. Im. S S Korsakova. 110, 12–19. 20559269

[B42] Sanderson C. M. Connell J. W. Edwards T. L. Bright N. A. Duley S. Thompson A.. (2006). Spastin and atlastin, two proteins mutated in autosomal-dominant hereditary spastic paraplegia, are binding partners. Hum. Mol. Genet. 15, 307–318. 10.1093/hmg/ddi447 16339213 PMC2443951

[B43] Solowska J. M. Garbern J. Y. Baas P. W. (2010). Evaluation of loss of function as an explanation for SPG4-based hereditary spastic paraplegia. Hum. Mol. Genet. 19, 2767–2779. 10.1093/hmg/ddq177 20430936 PMC2893808

[B44] Solowska J. M. Rao A. N. Baas P. W. (2017). Truncating mutations of SPAST associated with hereditary spastic paraplegia indicate greater accumulation and toxicity of the M1 isoform of spastin. Mol. Biol. Cell. 28, 1728–1737. 10.1091/mbc.e17-01-0047 28495799 PMC5491181

[B45] Stuchell-Brereton M. D. Skalicky J. J. Kieffer C. Karren M. A. Ghaffarian S. Sundquist W. I. (2007). ESCRT-III recognition by VPS4 ATPases. Nature 449, 740–744. 10.1038/nature06172 17928862

[B46] Tsang H. T. Edwards T. L. Wang X. Connell J. W. Davies R. J. Durrington H. J.. (2009). The hereditary spastic paraplegia proteins NIPA1, spastin and spartin are inhibitors of mammalian BMP signalling. Hum. Mol. Genet. 18, 3805–3821. 10.1093/hmg/ddp324 19620182 PMC2748891

[B47] Wang X. Shaw W. R. Tsang H. T. Reid E. O'Kane C. J. (2007). Drosophila spichthyin inhibits BMP signaling and regulates synaptic growth and axonal microtubules. Nat. Neurosci. 10, 177–185. 10.1038/nn1841 17220882 PMC2464677

[B48] White S. R. Evans K. J. Lary J. Cole J. L. Lauring B. (2007). Recognition of C-terminal amino acids in tubulin by pore loops in Spastin is important for microtubule severing. J. Cell Biol. 176, 995–1005. 10.1083/jcb.200610072 17389232 PMC2064084

[B49] Xie Y. Zhou B. Lin M.-Y. Wang S. Foust Kevin D. Sheng Z.-H. (2015). Endolysosomal deficits augment mitochondria pathology in spinal motor neurons of asymptomatic fALS mice. Neuron 87, 355–370. 10.1016/j.neuron.2015.06.026 26182418 PMC4511489

[B50] Yang D. Rismanchi N. Renvoise B. Lippincott-Schwartz J. Blackstone C. Hurley J. H. (2009). Structural basis for midbody targeting of spastin by the ESCRT-III protein CHMP1B. Nat. Struct. Mol. Biol. (2008) 15, 1278–1286. 10.1038/nsmb.1512 18997780 PMC2593743

[B51] Zehr E. Szyk A. Piszczek G. Szczesna E. Zuo X. Roll-Mecak A. (2017). Katanin spiral and ring structures shed light on power stroke for microtubule severing. Nat. Struct. Mol. Biol. 24:717. 10.1038/nsmb.3448 28783150 PMC7152510

