# Supplementary figures and images for: Spastin MIT Domain Disease-Associated Mutations Disrupt Lysosomal Function
Source: Front Neurosci. 2019 Nov 8;13:1179. doi: 10.3389/fnins.2019.01179 (PMC6856053; doi:10.3389/fnins.2019.01179)

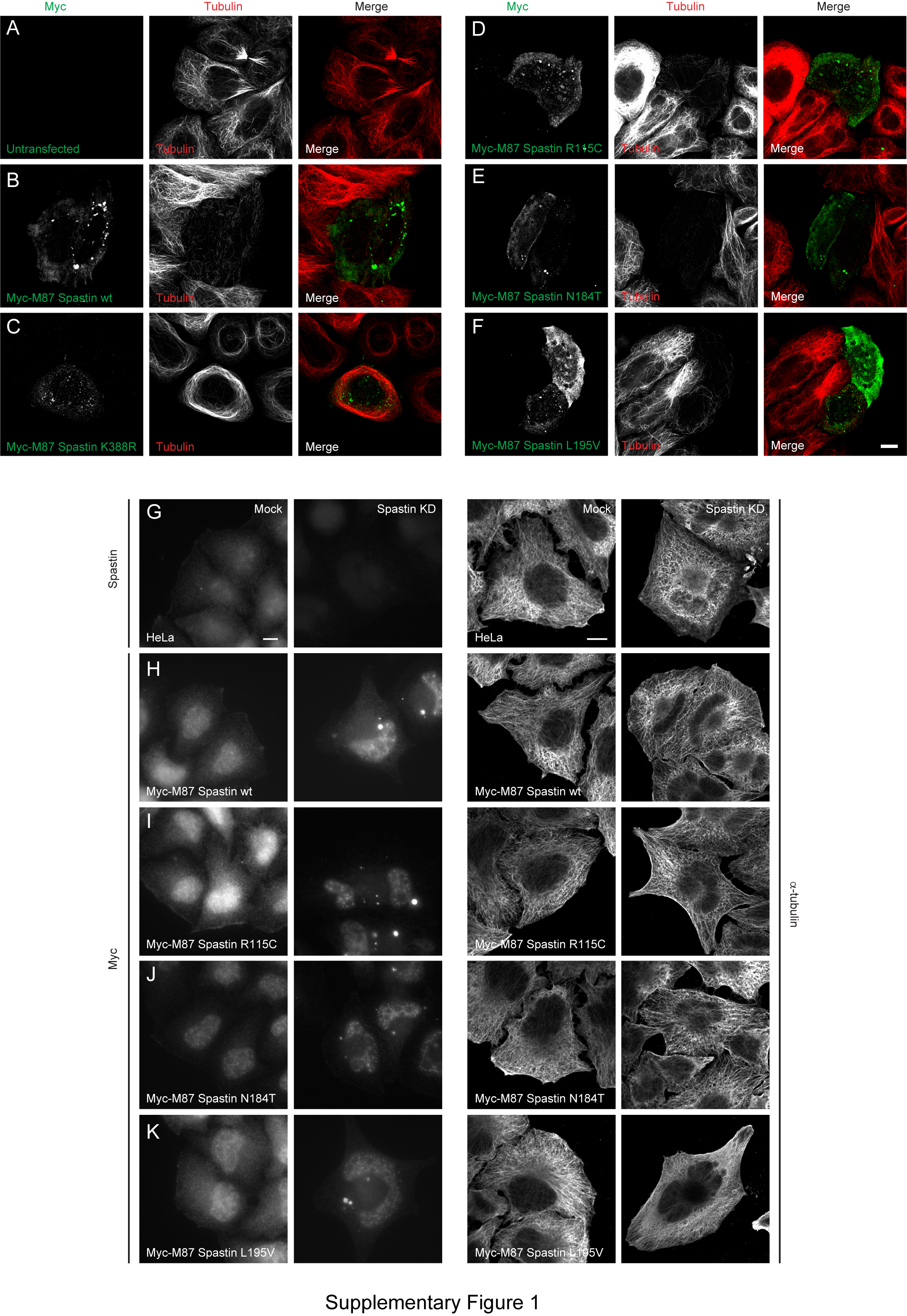

Supplement: Supplementary Figure 1 — Spastin's microtubule severing activity is unaffected by MIT mutants. (A–F) Wild-type Hela cells were transiently transfected with the myc-tagged M87-spastin constructs indicated. Cells were fixed and then processed for immunofluorescence microscopy and labeled with antibodies to myc and α-tubulin. Representative images showing the appearance of the microtubule architecture following spastin overexpression are shown. Scale bar = 10 μm. (G–K) Wild-type Hela cells or cells stably expressing the myc-tagged M87-spastin constructs indicated were fixed, then processed for immunofluorescence microscopy and labeled with (G) spastin and α-tubulin or (H–K) myc and α-tubulin. Representative images showing the appearance of microtubule cytoskeleton in each cell line, with and without endogenous spastin depletion, are shown. Scale bars = 10 μm. [file Image_1.TIF]

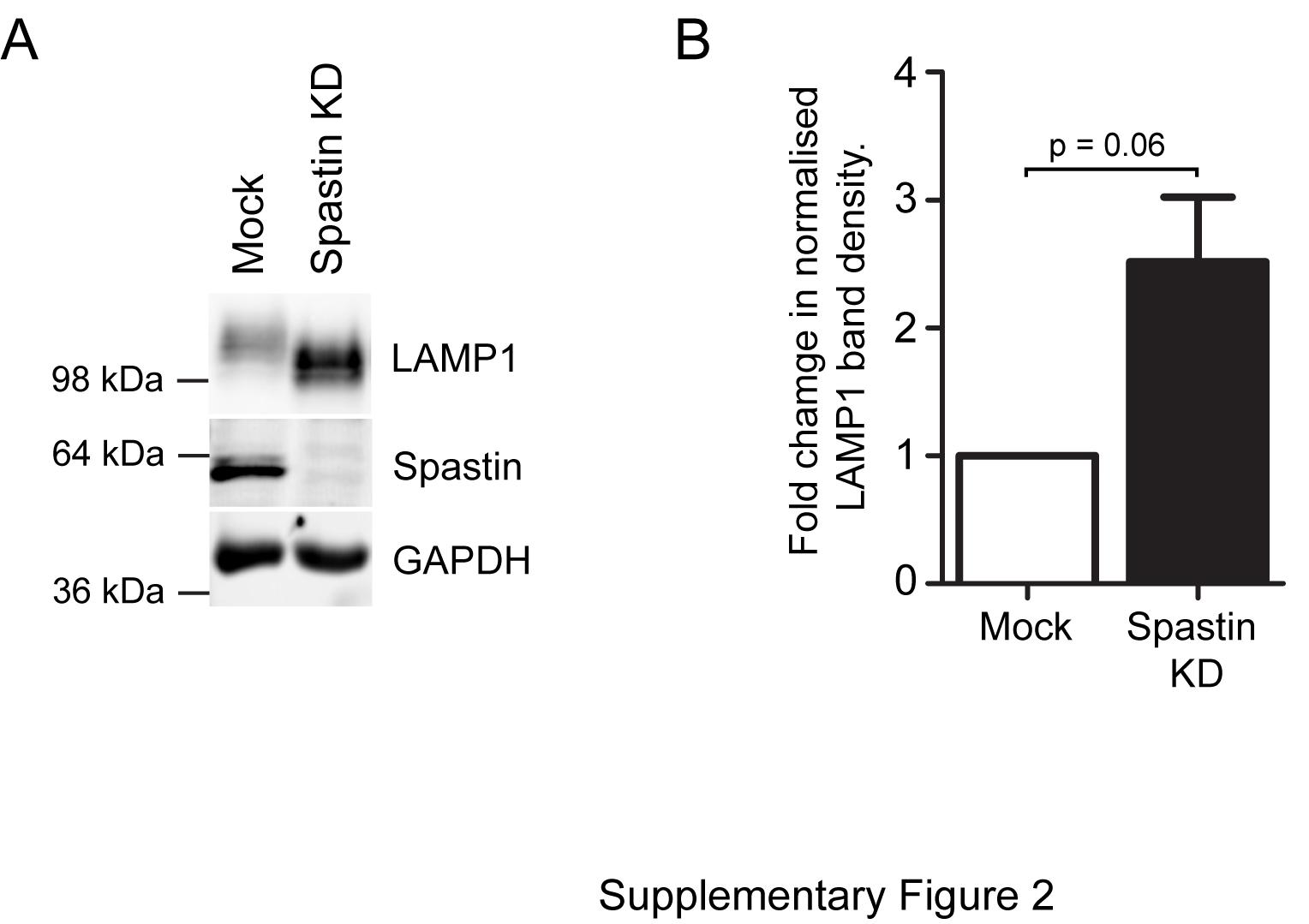

Supplement: Supplementary Figure 2 — Cells lacking spastin exhibit upregulation and altered migration of LAMP1. (A) HeLa cells were mock-transfected or subjected to spastin depletion by siRNA transfection, then immunoblotted with the antibodies shown. Note increased abundance and altered LAMP1 migration in the immunoblot from cells lacking spastin. Quantification of LAMP1 immunoblot band intensity is shown in (B), n = 4, p-values generated by paired t-tests. [file Image_2.TIF]
